# Secondary intraocular extranodal natural killer/T-cell lymphoma with optic nerve involvement: a case report

**DOI:** 10.3389/fonc.2026.1954795

**Published:** 2026-10-20

**Authors:** Sinan Ersan, Charles Zhang, Sofia M. Miguez, Maria A. Idarraga, Lubomir Sokol, Sander R. Dubovy, Carlos E. Mendoza-Santiesteban, Basil K. Williams

**Affiliations:** 1 Department of Ophthalmology, Storm Eye Institute, Medical University of South Carolina, Charleston, SC, United States; 2 Department of Ophthalmology, Ross Eye Institute, University at Buffalo, Buffalo, NY, United States; 3 Retina Consultants of Texas, Bellaire, TX, United States; 4 Department of Ophthalmology, Bascom Palmer Eye Institute, University of Miami Miller School of Medicine, Miami, FL, United States; 5 Florida Lions Ocular Pathology Laboratory, University of Miami Miller School of Medicine, Miami, FL, United States; 6 Department of Malignant Hematology, H. Lee Moffitt Cancer Center, Tampa, FL, United States

**Keywords:** intraocular lymphoma, lymphoma, natural killer/T-cell lymphoma, NK/T-cell, NKTL, optic nerve

## Abstract

A 61-year-old female with a history of extranodal natural killer/T-cell (NK/T-cell) lymphoma of the left thigh presented with a six-month history of presumed vitritis in the left eye managed by an outside provider. Diagnostic vitrectomy revealed atypical lymphocytes positive for CD3, CD7, CD56, and Epstein–Barr virus-encoded RNA, confirming the diagnosis of secondary extranodal intraocular NK/T-cell lymphoma. Initial systemic imaging showed no evidence of extraocular disease; nonetheless, systemic chemotherapy and external beam radiation therapy were recommended. The patient elected treatment with intravitreal methotrexate alone, with early improvement in visual acuity and intraocular inflammation. However, over the following three months, she developed retinal vasculitis and sub-retinal pigment epithelium infiltrates of the left eye, and abducens nerve palsy of the right eye. A week later, she developed acute worsening of symptoms in the left eye. MRI at the time demonstrated posterior optic nerve enhancement and nasal cavity involvement. Systemic and intrathecal chemotherapy was subsequently initiated. This represents a rare presentation of secondary intraocular extranodal NK/T-cell lymphoma with subsequent optic nerve involvement. The case highlights the aggressive clinical course and neurotropic potential of NK/T-cell lymphoma, underscoring the limitations of local therapy alone and the importance of early systemic evaluation and treatment.

## Introduction

Secondary intraocular lymphomas are rare and diagnostically challenging malignancies, often masquerading as infectious or inflammatory conditions (1). Approximately 74.3% of intraocular lymphomas are of B-cell lineage; in contrast, intraocular T-cell lymphomas are exceedingly rare, comprising only 1.3% of cases (1–4). Natural killer/T-cell (NK/T-cell) lymphomas represent an even rarer subset, accounting for <2% of all T-cell lymphomas (1). NK/T-cell lymphoma is an aggressive extranodal malignancy strongly associated with Epstein–Barr virus (EBV) (1). It most commonly involves the nasal cavity and upper respiratory tract. This disease is encountered predominantly in individuals from Asia and Latin America, suggesting that both genetic predisposition and geographic variation in EBV strains may play a central role in its pathogenesis (1, 5–7). Clinically, NK/T-cell lymphoma is characterized by its rapid progression, high propensity for tissue necrosis, and frequent dissemination to extranodal sites (1). When intraocular structures are involved, the diagnosis can be especially challenging due to the ability of NK/T-cell lymphoma to mimic other ocular conditions (1). Moreover, intraocular NK/T-cell lymphoma often indicates advanced systemic disease and is associated with a poor prognosis, underscoring the need for a high index of suspicion and prompt tissue diagnosis (1). Early recognition and multidisciplinary management are crucial, as delays in therapy can lead to irreversible vision loss and systemic progression.

Few cases have described an intraocular manifestation of systemic NK/T-cell lymphoma, most of which were due to spread from the adjacent nasal cavity (8–12). Even fewer cases were reported of intraocular spread from distant locations including the testes and retroperitoneum (12–14). Here, we present a rare case of NK/T-cell lymphoma of the left thigh with intraocular relapse and subsequent optic nerve involvement, highlighting the diagnostic complexity, systemic progression, and therapeutic considerations of this aggressive and uncommon malignancy.

## Case description

A 61-year-old female presented to the Bascom Palmer Eye Institute emergency department with a six-month history of progressive, painless vision loss in the left eye (OS). She was previously seen by an outside ophthalmologist who initiated difluprednate and atropine for suspected vitritis. Despite experiencing an initial improvement with these medications, she eventually noticed a gradual recurrence and worsening of vision loss.

The patient was diagnosed with EBV-positive extranodal NK/T-cell lymphoma based on a left thigh skin biopsy six months prior to presenting to the Bascom Palmer Eye Institute emergency department. Staging imaging with positron emission tomography/computed tomography (PET/CT) demonstrated stage IV disease, characterized by dominant fluorodeoxyglucose-avid cutaneous and subcutaneous masses with numerous additional smaller cutaneous nodules. Flow cytometric analysis of the bone marrow aspirate and peripheral blood demonstrated low-level involvement by NK/T-cell lymphoma. She received two cycles of SMILE-based chemotherapy (dexamethasone, methotrexate, ifosfamide, L-asparaginase, and etoposide), consisting of dexamethasone, methotrexate, ifosfamide, etoposide, and pegaspargase. Pegaspargase was omitted from the second cycle, which was completed approximately 3 months before presentation to the Bascom Palmer Eye Institute. Upon completion of chemotherapy, PET/CT demonstrated no evidence of metabolically active disease. Nevertheless, in accordance with contemporary guideline-based recommendations, her oncologists advised the administration of additional cycles of chemotherapy. Despite these recommendations, the patient chose not to pursue additional chemotherapy at that time, citing the normal imaging findings and her wish not to continue treatment. All other medical, social, and family histories were unremarkable, and the patient’s only medication was an over-the-counter multivitamin.

At presentation to the Bascom Palmer Eye Institute emergency department, visual acuity was 20/25 in the right eye (OD) and counting fingers at 2 ft OS. Intraocular pressure (IOP) was within normal limits, measuring 11 mm Hg OD and 14 mm Hg OS. The anterior segment examination was positive for 2+ nuclear sclerosis in both eyes (OU), 2+ cortical cataract OU, and trace cell OS. Dilated fundus examination was unremarkable OD but revealed vitritis OS (**Figure 1A**). B-scan ultrasonography OS demonstrated moderately dense, mobile vitreous opacities consistent with cells, veils and non-specific debris.

**Figure 1:**
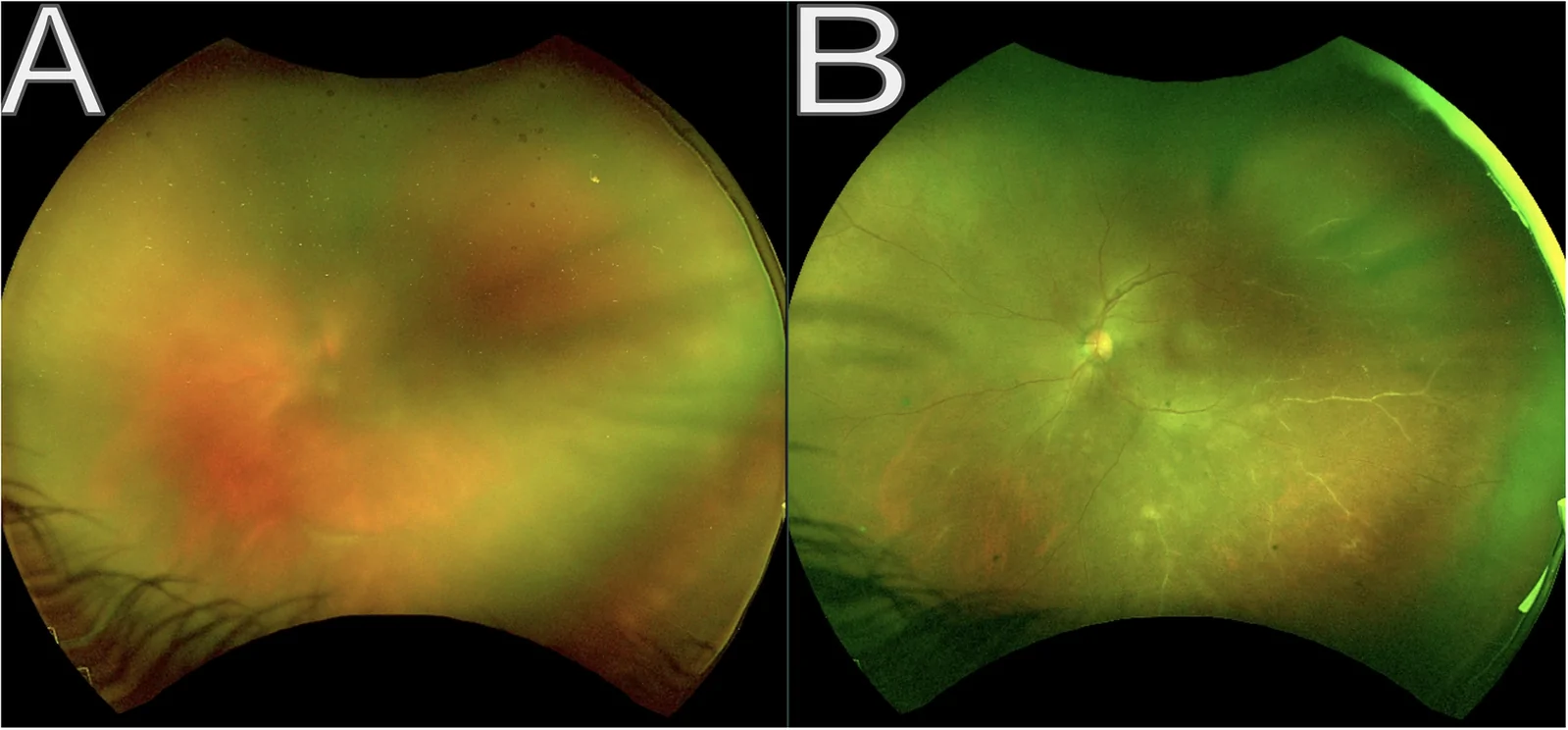
Ultra-widefield fundus photographs of the left eye at initial presentation demonstrating dense vitritis with poor visualization of the fundus **(A)**. At three weeks after the initial photograph and following pars plana vitrectomy, inferotemporal peripheral vessel whitening and sheathing consistent with retinal vasculitis were observed **(B)**.

Laboratory testing, including syphilis (rapid plasma reagin (RPR) and fluorescent treponemal antibody absorption (FTA-ABS) testing) and QuantiFERON-Tb Gold Plus, QIAGEN GmbH, Hilden, Germany, was negative. A diagnostic pars plana vitrectomy OS was performed, and cytopathologic examination demonstrated a diffuse infiltrate of atypical, moderately sized lymphoid cells with irregular nuclei, coarse chromatin, and scant cytoplasm. Immunohistochemical staining showed that the atypical cells were positive for CD3, CD7, and CD56 and negative for CD20. *In situ* hybridization for EBV-encoded RNA (EBER) was positive, confirming the presence of EBV within the tumor cells. The morphologic features and immunophenotypic profile were consistent with intraocular, extranodal NK/T-cell lymphoma.

The intraocular presentation of NK/T-cell lymphoma prompted a multidisciplinary discussion with the patient. An urgent repeat PET/CT scan was performed, which did not reveal any additional disease foci outside the eye. However, the care team recommended initiating systemic chemotherapy and external beam radiation therapy (EBRT) given the possibility of occult systemic or central nervous system (CNS) involvement. The patient declined both treatments due to the absence of detectable extraocular disease on imaging and concerns regarding potential treatment-related side effects. Despite understanding the associated risks, she maintained her decision, and additional treatment options were discussed; she ultimately elected to proceed with local therapy consisting of weekly intravitreal methotrexate injections (400 μg/0.1 mL).

At the follow-up visit after the first intravitreal methotrexate injection, visual acuity improved to 20/150 OS, and the vitreous had cleared. With improved visualization of the fundus, vessel whitening and sheathing in the inferotemporal periphery OS, which had not been present at the time of vitrectomy, were observed (**Figure 1B**). This retinal vasculitis was presumed to be secondary to the underlying lymphoma, but occlusive vasculitis secondary to methotrexate could not be completely ruled out. These findings were discussed with the patient, and systemic chemotherapy and EBRT were again recommended as the primary treatment approach. Alternative or adjunctive options, including local corticosteroids, continued intravitreal methotrexate, or observation, were also discussed. After reviewing the risks and benefits of each, the patient elected observation without further treatment.

A week later, optical coherence tomography (OCT) of the inferior mid-periphery OS demonstrated new sub-retinal pigment epithelium (sub-RPE) infiltrates, which were not seen on baseline OCT a few weeks prior (**Figures 2A, B**). After an additional discussion regarding available treatment options, and continued refusal by the patient to proceed with systemic chemotherapy and/or EBRT, intravitreal methotrexate injections were resumed. The patient was subsequently monitored closely with serial examinations and imaging. After an additional intravitreal methotrexate injection, resolution of the vasculitis was observed.

**Figure 2:**
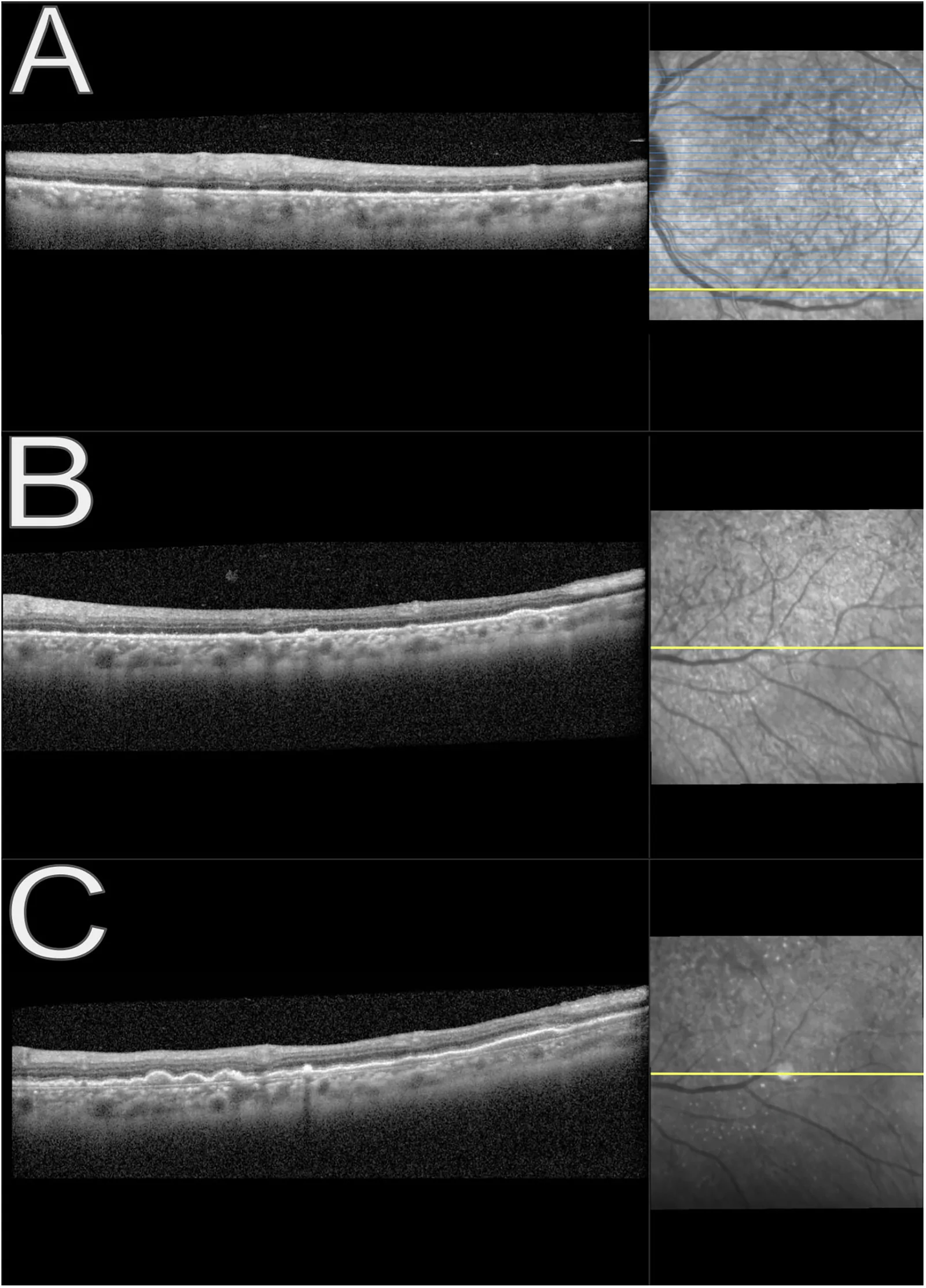
Optical coherence tomography of the left eye through the inferior arcade. Baseline imaging demonstrates irregular thickening of the retinal pigment epithelium (RPE) without sub-RPE infiltrates **(A)**. Imaging a few weeks later demonstrates development of sub-RPE infiltrates above the level of Bruch’s membrane **(B)**. At three months after the initial imaging, there is an interval increase in the extent and prominence of the sub-RPE infiltrates **(C)**.

A month later, 3 months after her initial presentation to the Bascom Palmer Eye Institute, the patient developed a new abducens nerve (CN VI) palsy OD, and the remainder of the clinical examination was within normal limits. By this time, the patient had received six total intravitreal methotrexate injections. Although treatment was initially planned for weekly administration, which would have resulted in double the number of injections during this period, the intended schedule was not maintained due to missed or delayed appointments. Visual acuity was 20/80 OS, but examination was severely limited by a 3+ posterior subcapsular cataract. Repeat OCT at the inferior arcade OS was obtained and showed worsening sub-RPE infiltrates (**Figure 2C**). Given the new-onset CN VI palsy OD, MRI of the brain was performed to evaluate for lymphomatous involvement of the orbit, nasal cavity, or CNS. No evidence of extraocular disease or CNS involvement was appreciated. Given the neurotropism of NK/T-cell lymphoma, initial imaging may be falsely negative in patients presenting with CN VI palsy; therefore, follow-up MRI was recommended.

A week later, the patient returned with an acute decline in vision OS to 20/400, accompanied by a new afferent pupillary defect. Repeat B-scan ultrasonography OS at this visit demonstrated moderate vitreous opacities, improved compared to prior imaging, along with a new finding of fullness of the optic nerve head. OCT of the retinal nerve fiber layer was subsequently performed and confirmed optic nerve head edema OS (**Figure 3**). A repeat MRI of the brain and orbits was obtained urgently, revealing an abnormal T2 hyperintense signal of the posterior intraorbital and intracanalicular left optic nerve and nerve sheath, and an enlarging T2 hypointense lesion in the posterior left nasal cavity. The remainder of the brain MRI was unremarkable, with no evidence of leptomeningeal enhancement, brain parenchymal lesions, or other intracranial involvement.

**Figure 3:**
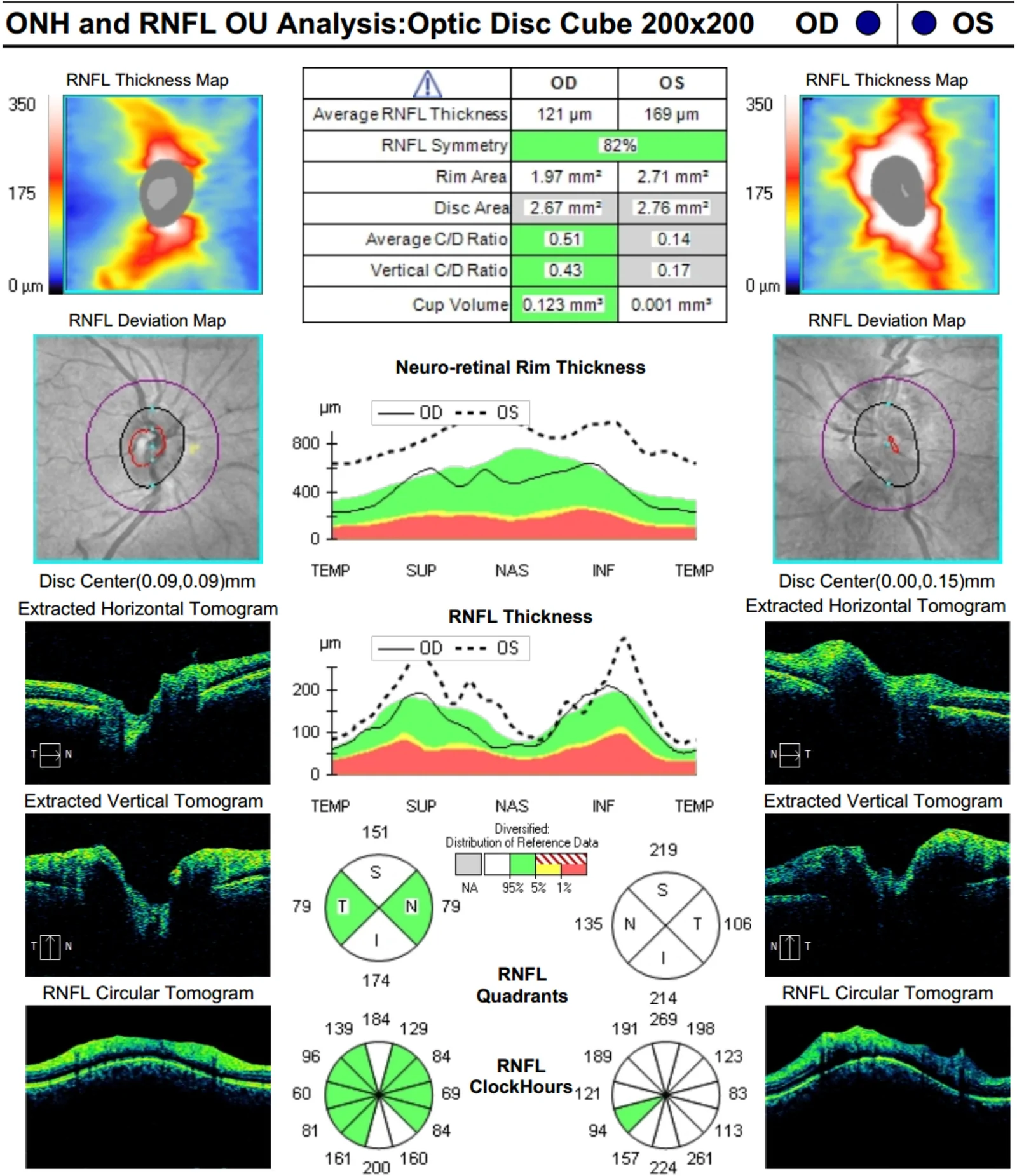
Optical coherence tomography of the retinal nerve fiber layer was normal in the right eye. The left eye shows optic nerve head edema with increased thickening of the neuroretinal rim and retinal nerve fiber layer.

Given the optic nerve and nasal cavity involvement of the lymphoma, the patient agreed to pursue more aggressive treatment. She received two additional intravitreal methotrexate injections, for a total of eight, and was started on systemic and intrathecal chemotherapy with dexamethasone, high-dose methotrexate, ifosfamide, L-asparaginase, and etoposide (SMILE– high-dose methotrexate (HD-MTX regimen)). After two cycles of chemotherapy, the patient reported notable improvement in systemic symptoms, and her CN VI palsy had improved. However, her visual acuity OS deteriorated to no light perception. Due to progression of the cataract, there was no view of the posterior segment at this time, and OCT to assess for resolution of sub-RPE infiltrates could not be performed. B-scan ultrasonography showed vitreous opacities, and the optic nerve head no longer appeared swollen.

Over the subsequent 16 months, the patient received two doses of intrathecal cytarabine. Cerebrospinal fluid cytologic and flow-cytometric analyses showed no evidence of malignant involvement. She subsequently completed four cycles of a modified dexamethasone (DDGP) regimen consisting of dexamethasone, cisplatin, and gemcitabine, with pegaspargase omitted. Following disease recurrence, she received an additional cycle of this regimen and was initiated on pembrolizumab. She ultimately underwent radiation therapy to the bilateral orbits, paranasal sinuses, and skull base, receiving a total dose of 36 Gy in 20 fractions.

At the final documented follow-up, approximately 23 months after initial presentation, the patient’s lymphoma remained in remission. Visual acuity was 20/30 OD, with an IOP of 10 mm Hg. Examination OD demonstrated a mild abduction deficit and moderate cataract, with no evidence of vitritis or subretinal infiltrates on dilated fundus examination. Visual acuity remained no light perception OS, with an IOP of 2 mm Hg. Examination OS demonstrated a shallow anterior chamber, 360 degrees of posterior synechiae, and a large white cataract that precluded visualization of the posterior segment. The patient’s clinical course is summarized in **Table 1**.

**Table 1:** Timeline of clinical course.

Time	Clinical event
6 Months Before presentation	• Diagnosed with EBV-positive stage IV extranodal NK/T-cell lymphoma following left thigh skin biopsy • Treated with two cycles of modified SMILE chemotherapy
0 to 6 Months Before Presentation	• Developed progressive painless vision loss OS • Treated for presumed vitritis with difluprednate and atropine at an outside facility
Week 0 (Presentation)	• Examination demonstrated vitritis OS • Diagnostic pars plana vitrectomy confirmed EBV-positive intraocular NK/T-cell lymphoma • PET/CT showed no evidence of extraocular disease
Week 1	• Declined recommended systemic chemotherapy and external beam radiation therapy • Initiated weekly intravitreal methotrexate • Following first injection, visual acuity improved and vitreous inflammation decreased, but new peripheral retinal vasculitis developed
Weeks 2 to 12	• OCT demonstrated new sub-RPE infiltrates OS • Retinal vasculitis resolved following an additional intravitreal injection of methotrexate • Developed a new abducens nerve palsy OD
Weeks 13 to 16	• Acute vision loss OS • Repeat MRI demonstrated lymphomatous involvement of the left optic nerve and a new lesion within the left nasal cavity. • Started systemic and intrathecal SMILE–HD-MTX chemotherapy. • After two cycles, systemic symptoms improved and the CN VI palsy resolved; however, vision OS deteriorated to NLP despite resolution of optic nerve edema and vitreous disease.
Weeks 17+	• Intrathecal cytarabine • Modified DDGP chemotherapy • Radiation therapy to bilateral orbits

Chronological summary of the patient's clinical presentation, diagnostic evaluation, treatment course, and disease progression from initial diagnosis of EBV-positive stage IV extranodal NK/T-cell lymphoma through the development of intraocular and optic nerve involvement, subsequent management, and clinical outcomes.

Abbreviations: CNS, central nervous system; DDGP, dexamethasone, cisplatin, gemcitabine, and pegaspargase; EBV, Epstein–Barr virus; HD-MTX, high-dose methotrexate; MRI, magnetic resonance imaging; NLP, no light perception; OCT, optical coherence tomography; OD, right eye; OS, left eye; PET/CT, positron emission tomography/computed tomography; RPE, retinal pigment epithelium; SMILE-HD-MTX, steroid (dexamethasone), high-dose methotrexate, ifosfamide, L-asparaginase, and etoposide.

## Discussion

NK/T-cell lymphoma was previously classified by the WHO as “extranodal NK/T-cell lymphoma, nasal type,” but is now termed “extranodal NK/T-cell lymphoma” to better capture the extent of extranodal disease outside the nasal cavity (15). Most reported cases of intraocular NK/T-cell lymphoma result from direct extension from the nasal cavity, theorized to occur due to close anatomic proximity. However, extranodal NK/T-cell lymphoma is an aggressive malignancy, so tumor cells arising at distant extranasal sites, including the testes, retroperitoneum, and skin or soft tissue, can spread systemically to the eye (8–14, 16). The current case demonstrates a rare scenario of intraocular involvement of NK/T-cell lymphoma that originated in the thigh and subsequently involved the nasal cavity and optic nerve.

The prognosis for patients with advanced-stage relapsed or refractory extranodal NK/T-cell lymphoma remains poor, with a median overall survival often <1 year and no universally accepted standard of care (17). Although consensus on optimal management is limited, evidence suggests that early intervention with combination chemotherapy regimens can be effective in inducing responses. Current National Comprehensive Cancer Network (NCCN) guidelines recommend starting a non-anthracycline-based chemotherapy regimen combined with L-asparaginase, such as SMILE-HD-MTX, typically consisting of four to six cycles, in all patients with advanced extranodal NK/T-cell lymphoma (18). In the current case, the patient was initially diagnosed with NK/T-cell lymphoma involving the left thigh and received two cycles of therapy. Despite medical recommendations, she elected to defer additional treatment due to the absence of disease on PET/CT imaging. Even after subsequent intraocular involvement and discussion of systemic re-treatment and EBRT, she again declined other forms of treatment in favor of local intravitreal methotrexate. This was despite counseling that a negative systemic PET/CT could not exclude occult systemic involvement. While aggressive treatment and patient compliance are critical to achieve the best outcomes in advanced NK/T-cell lymphoma, respect for patient autonomy is also critical (19). Nevertheless, intravitreal methotrexate injections were insufficient to prevent disease progression. It is likely that earlier initiation of systemic therapy and/or EBRT, followed by consolidation with allogeneic stem cell transplantation, may have altered the trajectory of the disease in this case.

Despite this, the current case offers important insights into the potential mechanisms of disease progression in NK/T-cell lymphoma when only local intraocular therapy is administered. It remains unclear whether invasion of the optic nerve occurred due to contiguous spread from intraocular lymphoma, secondary extension from occult CNS disease, or systemic reseeding of intraocular foci. The response to intravitreal methotrexate, as well as the temporal delay before the development of optic nerve and cranial nerve involvement, suggests a complex interplay between local control and systemic disease progression. As more cases of intraocular NK/T-cell lymphoma are reported and characterized, we may better understand the patterns of dissemination and the relative efficacy of local and systemic treatment strategies in managing this rare and aggressive malignancy.

## Data Availability

The original contributions presented in the study are included in the article/supplementary material. Further inquiries can be directed to the corresponding author.
